# Supplementary figures and images for: Large-Scale Meta-Longitudinal Microbiome Data with a Known Batch Factor
Source: Genes (Basel). 2022 Feb 22;13(3):392. doi: 10.3390/genes13030392 (PMC8953633; doi:10.3390/genes13030392)

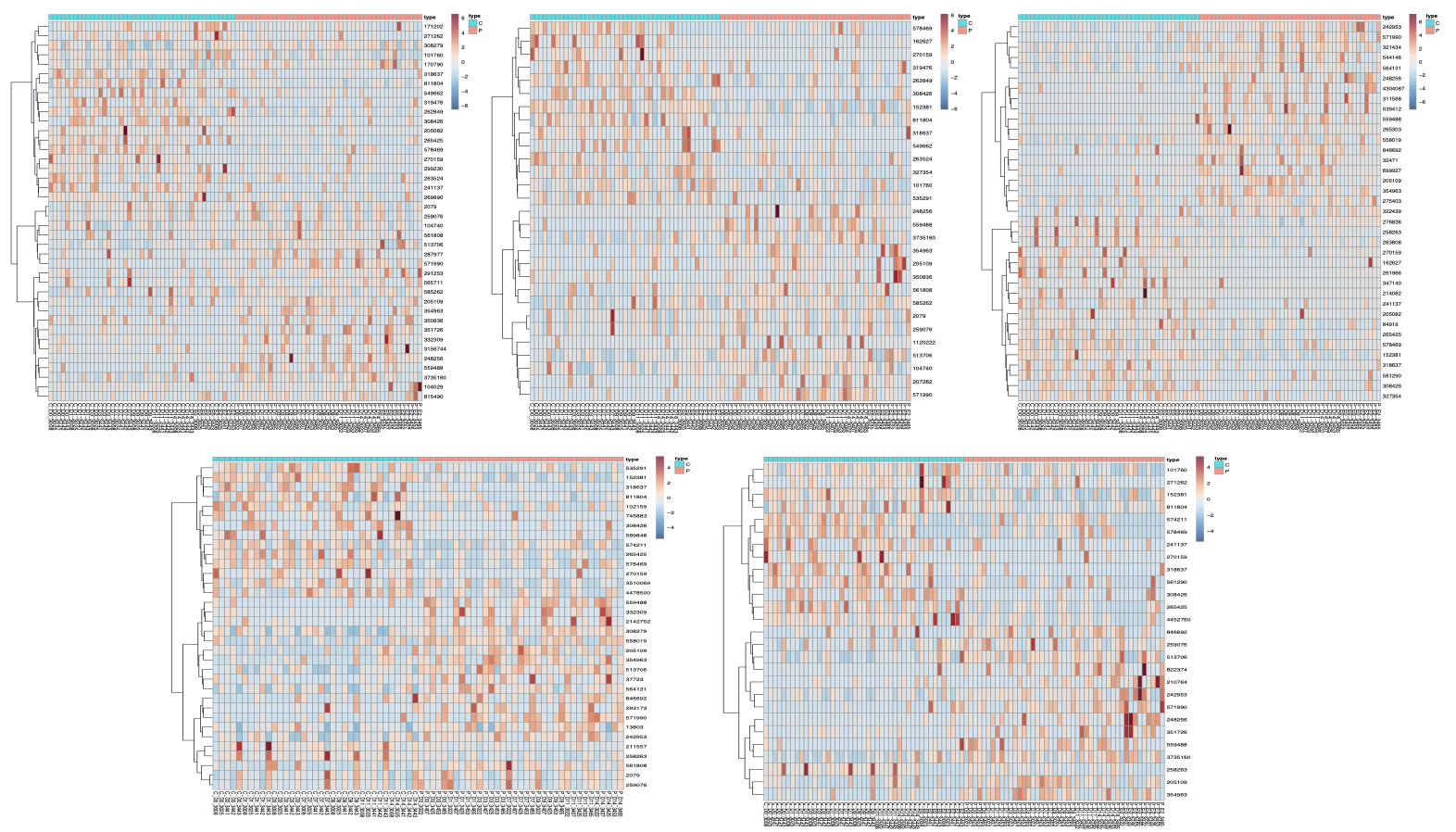

Supplement: Supplementary file 1 [file genes-13-00392-s001.zip › Supplementary Files/Supplementary Figure S1 -- Revision.png]

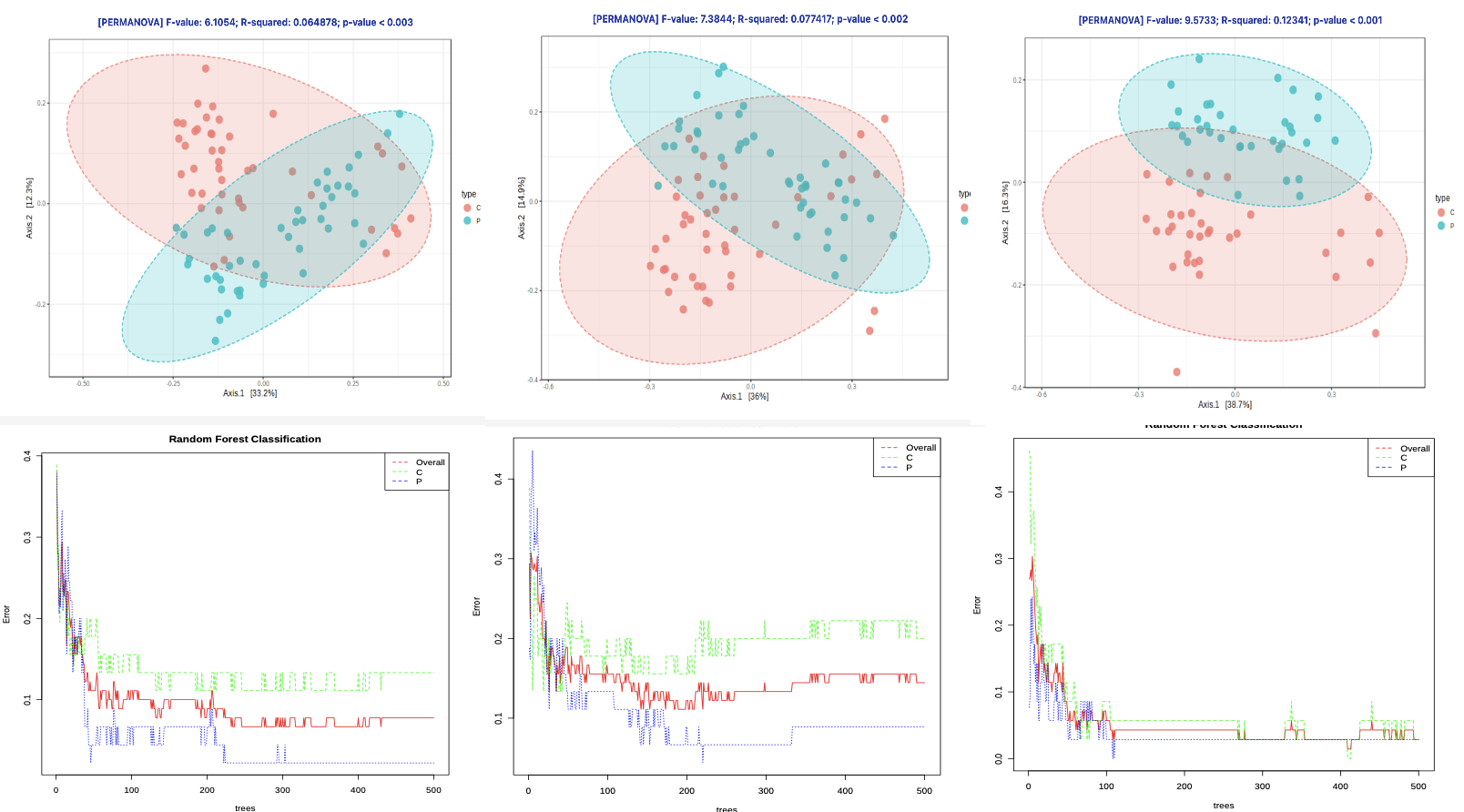

Supplement: Supplementary file 1 [file genes-13-00392-s001.zip › Supplementary Files/Supplementary Figure S2 -- Revision.png]
